# Factors associated with knowledge, attitudes and preventive practices towards COVID-19 in health care professionals in Lima, Peru

**DOI:** 10.12688/f1000research.53689.1

**Published:** 2021-07-16

**Authors:** Oriana Rivera-Lozada, Cesar Augusto Galvez, Elvis Castro-Alzate, Cesar Antonio Bonilla-Asalde

**Affiliations:** 1 Unidad de Posgrado de Salud Pública, Universidad Peruana Unión, LIMA, LIMA, LIMA 15, Peru; 2 VICERRECTORADO DE INVESTIGACIÒN, Universidad Norbert Wiener, LIMA, LIMA, LIMA 32, Peru; 3 South American Center for Education and Research in Public Health, Universidad Norbert Wiener, LIMA, LIMA, Peru; 4 Escuela de Rehabilitaciòn Humana, Universidad del Valle, CALI, VALLE DEL CAUCA, Colombia

**Keywords:** Health Knowledge, Attitudes and Practice; Health Personnel; Coronavirus infections; Peru

## Abstract

**Background:** Nowadays, we are facing a disease caused by SARS-CoV-2, known globally as COVID-19, which is considered a threat to global health due to its high contagiousness and rapid spread. **Methods:** Analytical cross-sectional study in 302 health professionals. An online questionnaire consisting of questions about knowledge, attitudes and practices (KAP) towards COVID-19 was applied. Socio-demographic, occupational and comorbidities factors were explored. Simple and multiple logistic regression models were used to identify factors associated with KAP. **Results:** Of the total, 25.2%, 31.5% and 37.4% had high levels of knowledge, preventive practices and risk perception attitudes respectively. Being married (aOR=6.75), having a master's degree (aOR= 0.41), having a working day with less than ten hours (ORa=0.49) and obesity (aOR=0.38) were associated with a low level of knowledge of COVID-19. The variables associated with preventive practices were being over the age of 50 (aOR=0.52), working in the hospitalization area (aOR=1.86) and having comorbidities such as arterial hypertension (aOR=0.28) and obesity (aOR=0.35). In relation to negative attitudes towards COVID 19, it was found that physical contact with patients with a confirmed diagnosis (aOR=1.84) and having asthma (aOR=2.13) were associated with these attitudes. **Conclusion:** Being married, having a master's degree, working less than ten hours were associated with having a low level of knowledge of COVID-19. Being older than 50, working in the hospitalization area were associated with preventive practices. Physical contact with COVID-19 patients was associated with negative attitudes.

## Introduction

We are currently facing a disease caused by the SARS-CoV-2 virus, known globally as coronavirus disease-2019 (COVID-19), which is considered a threat to global health due to its high contagiousness and rapid spread. ^1^ According to the World Health Organization (WHO), 159,896,332 cases of this disease and more than 3,321888 deaths were reported worldwide between its emergence in late 2019, and May 12, 2021. ^2^ Perú, like other Latin America countries, has been widely affected by the increase in confirmed cases. On May 12, 2021, the Ministry of Health (MINSA) reported 1,858,239 cases and 64,691 deaths, which makes Peru the nation with the sixth highest number of patients in the world. ^3^ However, there was a discrepancy between what was reported by MINSA and the figures reported by the National Death Registry Information System (Sistema Informático Nacional de Defunciones, SINADEF) since this institution reported 130,195 cases on the same date.

In Perú, health professionals are a population group at high risk of contracting COVID-19, because they are on the frontline in the fight against the disease. This disease is highly infectious; there is no specific treatment, and access to vaccination is still limited. ^4, 5^ Consequently, healthcare professionals must acquire sufficient knowledge to treat patients in an efficient and timely manner and, at the same time, protect themselves from contracting the disease.

It should be emphasized that, during the pandemic, healthcare professionals are being overworked in addition to feeling afraid of contracting the disease. ^6- 8^ This makes it more critical for any country to overcome the disease and protect healthcare professionals at the same time. ^7^

Consequently, a key element in the fight against the pandemic is to follow the guidelines established by organizations such as the World Health Organization (WHO) and MINSA. Given the challenges Perú is facing, adequate dissemination of information among health professionals is important so they can be updated with recent advances in managing the disease.

Hence, low levels of knowledge, attitudes and practices (KAP) in regard to the implementation of preventive measures against the disease ^2- 6, 9^ might cause serious public health problems, since health personnel must assume responsibility for care and control of the pandemic. ^7- 10^

Previous studies have reported that having low levels of knowledge, risk perception attitudes and preventive practices leads to a negative impact on the behavior towards the disease in health professionals. ^11- 16^ Therefore, it is essential to know what factors are associated with KAP to face COVID-19, providing evidence that is potentially useful for healthcare facilities to improve health interventions, which will reduce occupational exposure to COVID-19 in health professionals.

## Methods

### Study setting and design

The study used an analytical cross-sectional design. The sample population consisted of 302 health professionals who worked in healthcare facilities in Lima-Callao, and who also taught at the Faculty of Health Sciences of Norbert Wiener University, distributed across eight academic professional schools (APS) *(*Human medicine, Nursing, Obstetrics, Medical technology, Odontology, Human Nutrition, Psychology and Postgraduate School *)* in the second half of 2020. The instrument was administered in the August-December period, in 2020.

### Study population and size

The sample size was calculated probabilistically in two stages. In the first stage, the sample size was determined; in the second, the number of sample elements in each of the strata was calculated through proportional allocation. The sample size was divided by the population size and multiplied by the size of each stratum (APS). Thus, the size of the stratum was directly proportional to the sample size and the sampling was carried out by randomly selecting participants, using the list of health professionals of the academic schools that are part of the study population. Human medicine, Nursing and Obstetrics were the schools with the most representation, with 37.1% 14.4% and 14.3%, respectively.

To achieve our study objectives, we used the following selection criteria: health professionals working in a health facility in Lima-Callao who, additionally, were teaching at the faculty of Health Sciences or at the Postgraduate School of the Norbert Wiener University.

### Study procedure and tool

The questionnaire, described in the following pages, was validated by the judgment of ten experts, including pulmonologists, infectious disease specialists and epidemiologists, who determined their applicability to healthcare professionals in Perú.

The questionnaire to measure factors of interest had 20 questions that included sociodemographic factors (age, gender, marital status, number of children, level of education, religion and transportation), occupational factors (work area, working hours, contact with COVID-19 patients, relatives with suspected COVID-19 and physical contact with COVID-19 patients), comorbidity factors (diabetes, hypertension, asthma, cardiovascular diseases, obesity and overweight).

The competencies of health professionals on caring for COVID-19 patients were measured through their level of knowledge, preventive practices and risk perception attitudes. Regarding the level of knowledge on COVID-19, the WHO guidelines for clinical management of COVID-19 ^17^ and the questionnaire developed by Bhagavathula *et al.* ^18^ were considered. To this end, a survey of 44 questions was used to explore professionals’ knowledge on the disease’s etiology, symptoms, transmission, diagnosis, and prevention; the test score ranged from 0 to 44 points. These questions were answered on a true/false and “don’t know” basis. Correct questions scored one point and incorrect or unanswered answers scored zero; scores were converted into percentiles, a percentile ≥ 75% was categorized as high knowledge (33 or more correct answers) and <75% as low level of knowledge (fewer than 33 correct answers). The reliability of the questionnaire was 0.51, which was obtained through the use of the KR-20 to measure internal consistency, and is considered an accepted value to develop research processes. ^19^

Regarding the formulation of preventive practices-related questions, these were based on COVID-19 clinical management guidelines by WHO ^17^ and the Kim and Choi questionnaire. ^20^ Eleven questions considered practices such as hand washing, social distancing, surface disinfection, use of personal protective equipment, response to possible contagion. The answers were formulated on a Likert scale, which were subsequently recategorized into a “yes” or “no” dichotomous scale, where one point was assigned to an appropriate preventive practice and zero points to an inappropriate preventive practice. Scoring ranged from 0 to 11 points; a percentile ≥ 75% was categorized as high level of preventive practices (eight or more correct answers) and <75% as low level of preventive practices (fewer than eight correct answers). The instrument obtained a reliability coefficient of 0.78 through the KR-20 internal consistency index, and is therefore considered an acceptable level to develop research processes. ^19^

The attitude-related questions associated to risk perception were based on Zhang’s questionnaire, ^21^ which considered seven questions addressing factors such as confidence in defeating the virus, fear of infecting the family, concern that the equipment could not work, physical and mental exhaustion. The answers were formulated on a Likert scale and were subsequently ranked on a dichotomous “yes” or “no” scale. One point was assigned to an affirmative response and zero points to a negative response; scoring ranged from 0 to 7 points. A percentile ≥ 75% was categorized as high level of risk perception (five or more correct answers) and <75% as low level of risk perception (fewer than five correct answers). The questionnaire obtained a reliability coefficient of 0.77 using the KR-20 internal consistency index, and is considered an acceptable level to develop research processes. ^19^

Data collection was carried out through the distribution of an online questionnaire using Google Forms. Before filling out the questionnaire, everything was clearly and precisely explained via e-mail: the objectives of the study, voluntary participation, respect for confidentiality, the use of the obtained results and the description of the contact data. The surveys were anonymous and the data were treated with strict confidentiality; therefore, the completion of the questionnaires implied the informed consent of the professionals to participate in the study.

### Data management and analysis

Data analysis was performed in three phases. The first phase included descriptive analysis of the variables, using frequencies of the categorical variables. The second phase considered bivariate analysis, where the association between variables was evaluated by means of contingency tables, using odds ratios (OR) with their corresponding 95% CI confidence interval; for the statistical significance of the contingency tables, we used Fisher´s exact test when more than 20% of cells had expected frequencies < 5. Finally, in the third phase, a binary logistic regression analysis was performed to determine the factors associated with low levels of knowledge, risk perception attitudes and preventive practices toward COVID-19 infection in health professionals. The analyses were performed using SPSS version 26 (IBM) statistics program with a license provided by University of Valle (Cali, Colombia).

### Ethical considerations

Ethical standards were respected throughout the research process; the Institutional Research Ethics Committee of the Norbert Wiener University approved the study protocol and informed consent procedures with file No. -181-2020.

## Results

Information about 302 health professionals who were providing healthcare services during the period August-December 2020 was obtained. Regarding epidemiological variables, 64.9% were female and the median age was 46 years old (IQR 42-51), with greater participation of those under 50 old (73.5%). Regarding marital status, 87.4% (n = 264) were married or cohabiting, 7.0% (n = 21) were divorced and 5.6% (n = 17) were single, 91.4% (n = 276) had children. Regarding professions, 52.9% were physicians, 35.1% were nurses and 11.92% were obstetricians. The level of education corresponded to Master’s degree (79.1%), Doctorate (11.9%) and specialty (8.9%).

Regarding the area of work, the participants worked in outpatient consultation (32.8%), internal medicine department at the hospital (28.1%), intensive care unit (15.9%), emergency (13.9%) and clinical laboratory departments (9.3%). The median number of years of service was five (IQR 3-8) and the median daily working time was eight hours (IQR 7-8).

In the case of level of knowledge, it was established that 25.2% showed scores ≥ the 75th percentile, the parameter which allowed us to establish a high level of knowledge about COVID 19. Responses with the lowest scores were those related to the severity of the disease according to age groups (42.7%), time of subsistence of the virus (50%) and the need for specialized hospitals to care for suspicion or diagnosis of infection (55.6%).

In the case of preventive practices, 31.5% (n = 95) obtained scores above the 75th percentile, indicating a high level. A low level was identified in practices such as the use of disposable gloves in the workplace (45.0%), use of disposable gowns (42.1%), use of personal protective equipment (PPE) (25.2%) and decontamination of surfaces (7.7%).

The level of risk perception attitudes towards COVID 19 was analyzed with an inverse scale, determining the frequency of low levels of manifestation of negative attitudes (fear of contagion, fear that relatives might contract the disease, fear that personal protective equipment might not work, fear of death) like confidence, fear, concern and physical and mental fatigue. A total of 37.4% (n = 113) obtained scores above the 75th percentile, with a predominance of fear of becoming infected (49.7%), returning home and infecting the family (45%) and fear of dying from COVID 19 (49.7%).

Through a bivariate analysis, it was possible to establish that being married was a risk factor for having low levels of knowledge (OR = 7.01; CI: 1.64-29.85). The study showed, in addition, some preventive factors: having a Master’s degree (OR = 0.496; CI 0.27-0.90); working more than nine hours a day (OR = 0.36 CI: 0.16-0.75) and having relatives with diagnosed COVID-19 (OR = 0.47; CI 0.24-0.92).

Regarding preventive practices, it was shown that the use of public transport (OR = 1.68; CI 1.03-2.77), working in the hospital’s internal medicine department (OR = 2.11 CI 1.25-3.56) are risk factors for having a low level of preventive practices. However, we found some preventive factors such as being older than 50 (OR = 0.45; CI 0.24-0.83), experiencing comorbid conditions like hypertension (OR = 0.27; CI 0.08-0.94) and obesity (OR = 0.34; CI 0.14-0.79).

Regarding risk perception attitudes, the findings revealed risk factors such as having relatives with suspected COVID-19 (OR = 1.50; CI 1.08-2.64), having had contact with patients diagnosed with COVID-19 (OR = 1.92; CI 1.05-3.08) and having asthma as a comorbid condition (OR = 2.29; CI 1.17-4.50) ( Tables 1 and 2).

**Table 1.** Association between epidemiological variables and level of knowledge, practices and negative attitudes.

Epidemiological Variables	Knowledge				Practices				Negative attitudes			
	Low level (%, n)	High level (%, n)	OR (95% CI)	*P*	Low level (%, n)	High level (%, n)	OR (95% CI)	*P*	Low level (%, n)	High level (%, n)	OR (95% CI)	*P*
**Gender**												
Male	33.6 (76)	39.5 (30)	1 0.77 (0.45-1.13)	0.356	32.4 (67)	41.1 (39)	1 0.68 (0.41-1.13)	0.142	37.6 (71)	31.0 (35)	1 1.34 (0.81-2.20)	0.245
Female	66.4 (150)	60.5 (46)			67.6 (140)	58.9 (56)			62.4 (118)	69 (78)		
**Age**												
35 – 49 years old	74.3 (168)	71.1 (54)	1 1.18 (0.66-2.1)	0.575	69.1 (143)	83.2 (79)	**1** **0.45** (0.24-0.83)	**0.01**	70.9 (134)	77.9 (88)	1 0.69 (0.40-1.19)	0.18
50 – 65 years old	25.7 (58)	28.9 (22)			30.9 (64)	16.8 (16)			29.1 (55)	22.1 (25)		
**Marital status** *												
Single/cohabiting	15.9 (36)	2.6 (2)	1 7.01 (1.64-29.85)	**0.001**	11.1 (23)	15.8 (15)	0.66 (0.33-1.34)	0.255	13.2 (25)	11.5 (13)	1.17 (0.57-2.39)	0.66
Married/ cohabiting	84.1 (190)	97.4 (74)			88.9 (184)	84.2 (80)			86.8 (164)	88.5 (100)		
**Children**												
No	10.2 (23)	3.9 (3)	1 2.75 (0.80-9.45)	0.104	7.7 (16)	10.5 (10)	0.71 (0.31-1.6)	0.42	6.8 (15)	15.2 (11)	0.79 (0.35-1.80)	0.59
Yes	89.8 (203)	96.1 (73)			91.6 (191)	91.0 (85)			93.2 (189)	84.8 (113)		
**Level of education**												
Specialty	7.1 (16)	14.5 (11)	1		11.6 (24)	3.2 (3)	1		10.6 (16)	3.0 (11)	1	
Master	82.3 (186)	69.7 (53)	**0.496** (0.27-0.90)	**0.020**	78.3 (162)	81.1 (77)	1.18 (0.64-2.18)	0.57	77.1 (150)	86.4 (89)	0.96 (0.54-1.70)	0.90
Doctorate	10.6 (24)	15.8 (12)	1.57 (0.74-3.33)	0.229	10.1 (21)	15.8 (15)	1.61 (0.81-3.38)	0.16	12.3 (22)	10.6 (13)	0.94 (0.45-1.93)	0.86
**Religion**												
Non-Catholic	19.0 (43)	22.4 (17)	1 0.815 (0.43-1.53)	0.528	19.3 (40)	21.1 (20)	1 0.89 (0.49-1.64)	0.727	22.8 (43)	15.0 (17)	1 1.66 (0.89-3.08)	0.10
Catholic	81.0 (183)	77.6 (59)			80.7 (167)	78.9 (75)			77.2 (146)	85.0 (96)		
**Transport**												
Private	63.3 (143)	59.2 (45)	1 1.18 (0.69-2.01)	0.527	**66.2** **(137)**	**53.7** **(51)**	**1** **1.68** **(1.03-2.77)**	**0.037**	62.3 (119)	62.1 (69)	1 1.08 (0.67-1.75)	0.74
Public	36.7 (83)	40.8 (31)			33.8 (70)	46.3 (44)			37.7 (70)	37.9 (44)		

* *Fisher exact test*. The numbers in bold represent measures of association (Odds Ratio and statistical significance *p* < 0.05).

**Table 2.** Association between work variables and comorbidity conditions with level of knowledge, practices and negative attitudes.

Occupational factors	Knowledge				Practices				Negative attitudes			
	Low level (%, n)	High level (%, n)	OR (95% CI)	*P*	Low level (%, n)	High level (%, n)	OR (95% CI)	*P*	Low level (%, n)	High level (%, n)	OR (95% CI)	*P*
**Work area**												
Outpatient consultation	29.6 (67)	42.1 (32)	1		37.7 (78)	22.1 (21)	1		28.0 (53)	40.7 (46)	1	
Emergency	15.9 (36)	7.9 (6)	0.45 (0.18-1.12)	0.08	13.0 (27)	15.8 (15)	1.25 (0.63-2.47)	0.52	16.4 (31)	9.7 (11)	0.55 (0.26-1.14)	0.105
Hospitalization in the internal medicine department	27.4 (62)	30.3 (23)	1.14 (0.64-2.03)	0.635	23.2 (48)	38.9 (37)	**2.11** **(1.25-3.56)**	**0.005**	29.6 (56)	25.7 (29)	0.82 (0.48-1.38)	0.45
Laboratory department	9.3 (21)	9.2 (7)	0.99 (0.40-2.43)	0.98	10.1 (21)	7.4 (7)	0.70 (0.29-1.7)	0.44	10.1 (19)	8.0 (9)	0.40 (0.33-1.77)	0.54
ICU	17.7 (40)	10.5 (8)	0.55 (0.24-1.23)	0.139	15.9 (33)	15.8 (15)	0.98 (0.51-1.92)	0.97	15.9 (30)	15.9 (18)	1.004 (0.53-1.89)	0.99
**Working years**												
2-5 years	48.7 (110)	55.3 (42)	1		50.2 (104)	50.5 (48)	1		50.3 (95)	50.4 (57)	1	
6 – 10 years	32.3 (73)	23.7 (18)	0.65 (0.36-1.18)	0.157	28.5 (59)	33.7 (32)	1.27 (0.76-2.14)	0.36	30.7 (58)	29.2 (33)	0.93 (0.56-1.55)	0.78
More than 11 years	19.0 (43)	21.1 (16)	1.13 (0.59-2.16)	0.7	21.3 (44)	15.8 (15)	0.69 (0.36-1.32)	0.26	19.0 (36)	20.4 (23)	1.08 (0.60-1.94)	0.78
**Working hours**												
Up to four hours	2.2 (5)	7.9 (6)	1		1.6 (3)	7.2 (8)	1		1.6 (3)	7.1 (8)	1	
Up to eight hours	70.4 (159)	80.3 (61)	1.71 (0.91-3.22)	0.09	74.9 (155)	68.4 (65)	0.72 (0.42-1.08)	0.24	72.5 (137)	73.5 (83)	1.05 (0.62-1.77)	0.85
More than nine hours	27.4 (62)	11.8 (9)	**0.36** (0.16-0.75)	**0.006**	23.7 (49)	23.2 (22)	0.97 (0.54-1.72)	0.92	25.9 (49)	19.5 (22)	0.69 (0.39-1.21)	0.2
**Relatives diagnosed with COVID-19**												
No	69.9 (158)	82.9 (63)	1	0.027	72.5 (150)	74.7 (71)	1	0.67	77.2 (147)	66.4 (75)	1	0.039
Yes	84 (68)	16 (13)	0.47 (0.24-0.92)		27.5 (57)	25.3 (24)	0.89 (0.51-1.54)		22.8 (43)	33.6(38)	1.72 (1.03-2.88)	
**Relatives with suspected COVID-19**												
No	78.3 (177)	82.9 (63)	1	0.393	76.3 (158)	86.3 (82)	1	**0.046**	82.0 (155)	75.2 (85)	**1**	**0.015**
Yes	21.7 (49)	20.9 (13)	0.74 (0.38-1.46)		26.2 (49)	10.8 (13)	0.511 (0.26-0.99)		18.0 (34)	24.8 (28)	**1.50** **(1.02-2.64)**	
**Contact with COVID-19 patients**												
No	32.3 (73)	28.9 (22)	1	0.586	33.8 (70)	26.3 (25)	1	0.192	34.4 (65)	26.5 (30)	1	0.156
Yes	67.7 (153)	71.1 (54)	1.17 (0.66-2.06)		66.2 (137)	73.7 (70)	1.43 (0.83-2.45)		65.6 (124)	73.5 (83)	1.45 (8.67-2.42)	
**COVID-19 patient admission**												
No	65.0 (147)	59.2 (45)	1	0.361	66.7 (138)	56.8 (54)	1	0.099	66.1 (125)	59.3 (67)	1	0.23
Yes	35.0 (79)	40.8 (31)	1.28 (0.75-2.18)		33.3 (69)	43.2 (41)	1.52 (0.92-2.49)		33.9 (64)	40.7 (46)	1.34 (0.83-2.17)	
**Visual contact**												
No	38.9 (88)	35.5 (27)	1	0.596	40.1 (83)	33.7 (32)	1	0.309	41.3 (78)	32.7 (37)	1	0.14
Yes	61.1 (138)	64.5 (49)	1.157 (0.67-1.98)		59.9 (124)	66.3 (63)	1.32 (0.79-2.19)		58.7 (111)	67.3 (76)	1.44 (0.88-2.35)	
**Physical contact**												
No	54.4 (123)	47.4 (36)	1	0.287	55.6 (115)	46.3 (44)	1	0.135	58.7 (111)	42.5 (48)	**1**	**0.006**
Yes	45.6 (103)	52.6 (40)	1.32 (0.78-2.23)		44.4 (92)	53.7 (51)	1.44 (0.89-2.35)		41.3 (78)	57.7 (113)	**1.92** **(1.20-3.09)**	
**Contact with surface**												
No	54.0 (122)	61.8 (47)	1	0.232	59.4 (123)	48.4 (46)	1	0.074	60.3 (114)	48.7 (55)	1	0.166
Yes	46.0 (104)	38.2 (29)	0.724 (0.42-1.23)		40.6 (84)	51.6 (49)	1.56 (0.96-2.54)		39.7 (75)	51.3 (58)	1.60 (1.002-2.6)	
**Contact with suspected COVID-19**												
No	31.0 (70)	25.0 (19)	1	0.323	29.0 (60)	30.5 (29)	1	**0.78**	33.9 (64)	22.1 (25)	1	0.03
Yes	69.0 (156)	75 (57)	1.34 (0.74-2.43)		71.0 (147)	69.5 (66)	**0.93** (0.54-1.57)		66.1 (125)	77.9 (88)	1.8 (1.05-3.08)	
**Comorbidity**												
None	58.0 (131)	69.7 (53)			54.6 (113)	74.7 (71)	1		64.0 (121)	55.8 (63)	1	
Asthma	15.0 (34)	7.9 (6)	0.48 (0.19-1.20)	0.112	14.0 (29)	11.6 (11)	0.80 (0.38-1.68)	0.563	9.5 (18)	19.5 (22)	2.29 (1.17-4.50)	**0.014**
Diabetes	3.1 (7)	-	-	-	1.9 (4)	3.2 (3)	1.65 (0.36-7.54)	0.682	2.1 (4)	2.7 (3)	1.26 (0.27-5.74)	0.524
Hypertension	6.6 (15)	13.2 (10)	2.13 (0.91-4.96)	0.074	10.6 (22)	3.2 (3)	0.27 (0.080-0.94)	0.041	6.3 (12)	11.5 (13)	1.91 (0.84-4.36)	0.116
Obesity	17.3 (39)	9.2 (7)	0.48 (0.21-1.13)	0.09	18.8 (39)	7.4 (7)	0.34 (0.14-0.79)	0.010	18.0 (34)	10.6 (12)	0.54 (0.26-1.09)	0.085

The numbers in bold represent measures of association (Odds Ratio and statistical significance *p* < 0.05).

### Predictors of level of knowledge, preventive practices and negative risk perception attitudes towards COVID 19

Logistic regression analysis identified that being married (adjusted OR = 6.75, 95%CI 1.46-31.2) was a risk factor for a low level of knowledge of COVID 19. Preventive factors, such as having completed a Master’s degree (adjusted OR = 0.41, 95%CI 0.21-0.80), working more than 9 hours a day (adjusted OR = 0.49, 95%CI 0.25-0.95), presenting with obesity as a comorbidity condition (adjusted OR = 0.38, 95%CI 0.15-0.95) were also found. Multivariate analysis allowed us to estimate a coefficient of determination of 0.16, which explained 16% of the variance of the level of knowledge.

In relation to preventive practices, it was found that working in the hospital’s internal medicine department (adjusted OR = 1.86, 95%CI 1.08-3.18) was a predictor variable of risk for low level of preventive practices. In addition, protective factors such as being older than 50 (adjusted OR = 0.52, 95%CI 0.27-0.98), presenting with comorbidities such as hypertension (adjusted OR = 0.28, 95% CI 0.08-0.99) and obesity (adjusted OR = 0.35, 95%CI 0.14-0.83) were found. Multivariate analysis allowed us to estimate a coefficient of determination of 0.19, which explained 19% of the variance in the level of preventive practices.

Finally, regarding risk perception attitudes towards COVID-19, physical contact with patients with a confirmed diagnosis (adjusted OR = 1.84, 95%CI 1.14-2.97) and presenting with asthma as a comorbidity condition (adjusted OR = 2.13, 95%CI 1.081-4.22) were found as predictor variables. Multivariate analysis allowed us to estimate a coefficient of determination of 0.23, which explained 23% of the variance in the level of risk perception attitudes ( Table 3).

**Table 3.** Predictors of level of knowledge, preventive practices and negative attitudes towards COVID 19.

Part A. Regression model for knowledge				
Variable	Wald Statistic	Degrees of freedom	OR (95%CI)	*p*
Marital status Married/cohabiting	10.095	1	6.75 (1.46 – 31.2)	0.014
Level of education Master	6.312	1	0.41 (0.21 – 0.80)	0.009
Working hours, a day More than nine hours	6.525	1	0.49 (0.25 – 0.95)	0.036
Comorbidity Obesity	1.689	1	0.38 (0.15 – 0.95)	0.039
Constant	0.553	1	0.336	≤ 0.001

Part B. Regression model for practices				
Variable	Wald Statistic	Degrees of freedom	OR (95% CI)	*p*
Age Older than 50	3.127	1	0.52 (0.27 – 0.98)	0.0077
Work area Hospitalization	5.57	1	1.86 (1.08 – 3.18)	0.018
Comorbidity Arterial hypertension	5.43	1	0.28(0.081 – 0.99)	0.02
Comorbidity Obesity	5.497	1	0.35 (0.14 – 0.83)	0.019
Constant	-1.456	1	0.459	≤ 0.001

Part C. Model for attitudes				
Variable	Wald Statistic	Degrees of freedom	OR ( 95%CI)	*p*
Contact with patients with confirmed COVID-19	6.228	1	1.84 (1.14– 2.97)	0.006
Comorbidity Asthma	5.807	1	2.13 (1.081 – 4.22)	0.029
Constant	0.536	1	0.598	≤ 0.001

## Discussion

Our study revealed that healthcare professionals in Perú have insufficient knowledge about COVID-19 (more than 70% did not have a high level of knowledge), in contrast to a study in Nigeria, ^22^ where fewer than 20% of health professionals showed insufficient knowledge. Although frontline healthcare staff are expected to have a high level of knowledge of SARS-CoV-2, our study found a large knowledge gap regarding the severity of the disease according to age group and duration of virus persistence. Knowledge of the severity of the disease according to age group represents a weak link in clinical management, since therapeutic management is prioritized according to the risk of contracting a disease or its complications. ^23^ Regarding the persistence of the SARS-CoV-2 virus, it is important to highlight it can survive at least 72 hours on plastic surfaces and stainless steel. ^24^ This is fundamental in the prevention of person-to-person or patient-to-healthcare worker transmission during clinical care.

The present study revealed that being married represents a higher probability of having a low level of knowledge. Authors such as Naser *et al.*, ^25^ and Rani *et al*., ^26^ have shown associations between marital status and low levels of knowledge of COVID-19 in health professionals in Saudi Arabia, where low levels of knowledge were found in single health professionals, as opposed to married health professionals, which can be explained by cultural aspects of Eastern countries such as believing that children and young adults are at a lower risk of contracting the disease, attending crowded places such as markets and mosques, in addition to their low acceptance of the use of masks. ^25, 26^ These results are different from what we found in our study, where a low level of knowledge in married health professional was shown, which can be explained by the fact that the proportion of single population was low (12.6%).

In addition, regarding the methodological aspects of the present study, one factor that may affect the results is the low participation of single people under 40 to the study, which corresponds to the age at which continuous or post-graduate training processes are carried out.

However, this association was not observed in the level of practices and attitudes. This could be due to social reasons, as married people might have less time to do COVID-19 training courses, unlike single people who might have more free time to acquire such knowledge. However, the level of practices and attitudes would not change, which could be due to the experience acquired in healthcare.

It was found that some factors such as having a Master’s degree, working more than nine hours and having relatives diagnosed with COVID-19 were preventive factors against having a low level of knowledge. This could be happening because self-learning, such as that employed when studying for a Master’s program, plays a key role in the process of acquiring COVID-19 knowledge. Similar studies in physicians found that younger physicians and those who had not worked with patients for a long time had lower COVID-19 knowledge scores. ^27^ Presenting with comorbidity conditions was associated with good levels of knowledge, attitudes and practices towards COVID-19, which may be due to the fact that being part of a population at risk demands a greater level of care and attention to this disease compared to other groups that are not at risk. ^28^ The presence of comorbidity conditions contributes to the inclusion of self-care behaviors by health professionals, based on their personal and professional experience and, thus, they can minimize the risk of contagion in their workplace.

Studies conducted in some Asian countries found that health professionals had a high level of knowledge of COVID 19, but had low levels of preventive practices, which allows them to affirm that knowledge is not a determining factor in developing preventive practices and attitudes, and that other measures should be implemented, such as improvement of the work environment and access to adequate PPE. ^28, 30- 32^ In our research, it became evident that 75% had low levels of knowledge and preventive practices, despite the fact that about six months had passed since the notification of the first case of COVID 19 in Perú. The explanation for this situation could be related to the fact that much of the information on the pandemic circulating in the academic media came from the opinion of “experts”, social networks or the media, which lacked scientific rigor.

It is known that healthcare professionals who have received instructions on donning and discarding PPE could cause a decrease in the risk of making errors, as along with professionals who have had active training with spoken instructions and computer simulation on correct PPE removal. ^33^ A study in Jordan found that there was an association between biosafety at work and good biosafety practice at home, with a biosafety score at work of 73% (considered low by the researchers). ^31^ The only way to control new potentially deadly epidemics such as the one we are experiencing, and from an early stage, is to educate the population and especially healthcare personnel to adopt optimal behavior of biosafety practices and maximum PPE protection. ^34, 35^

In relation to preventive practices, we could identify an association with epidemiological variables such as age, i.e. being older than 50. This suggests that an increase in knowledge may lead to better attitudes and practices. In this case, it is known that COVID-19 affects people of any age, but people over 60 are more severely affected, ^36^ which may imply that older healthcare professionals, knowing that they are a population at a higher risk of contracting this disease, may follow better recommendations regarding preventive practices against COVID-19. Similarly, with respect to occupational factors, an association with being part of the hospital personnel was identified; a possible explanation may be that due to the serious clinical conditions of patients with COVID-19 in hospitals, the involved physicians and health personnel made greater efforts to have preventive practices against contagion.

In the present study, we found that certain groups of medical professionals have little knowledge about COVID-19, which is why the importance of ensuring the delivery of knowledgeable information to medical professionals should be emphasized. These low levels of knowledge would explain why Perú has one of the highest rates of medical professionals infected with COVID-19. This should be taken into account by front line care teams, physician managers and, in general, all health professionals in order to eliminate knowledge gaps and improve COVID-19 knowledge scores, attitudes and practices.

Knowledge allows the establishment of prevention strategies to avoid the spread of the virus, and also facilitates the development of positive attitudes towards the acquisition of self-care habits at work as well as respect for the rights of patients diagnosed with COVID-19, and the recognition of the effectiveness of the treatment plan and coping behaviors. ^37^ In addition, exposure to the virus in the workplace implies a mental burden and could have a negative impact on control measures, ^38, 39^ which increases the risk of infection. In the present study, among the risk perception attitudes, fear of becoming infected predominated, which coincides with the findings of Zhang *et al.*, Abdel *et al.*, and Maleki *et al.*, ^39, 41^ who found that between 85% and 92% of healthcare workers expressed fear of transmitting the disease to their family members. Therefore, it can be concluded that the perception of risk is a determining factor for the modification of attitudes in the work environment and the restructuring of healthy and safe behaviors during the working day, ^38, 39^ which impacts on family and social relations.

These results contrast with the findings of Abdelhafiz *et al.*, ^43^ who stated that stigma associated with the disease is based on fear associated with mortality and its transmission capacity. This could explain the association between the level of negative attitudes in those with relatives with suspected COVID-19, and having had contact with patients diagnosed with COVID-19. Although it may seem irrelevant, stigma is important because it can lead to public reluctance to seek medical care and the underreporting of cases, which can influence the increase in confirmed cases in a scenario characterized by community transmission. Thus, to combat stigma, it is necessary to develop appropriate education strategies framed in health policies and launching de-stigmatization programs in hospitals. ^43^

The main limitation of this study was that the attitudes and practices of health professionals may be overestimated, as they may answer interview questions in a way that they believe is socially acceptable rather than completely accurate, because of “social desirability”. ^44, 45^ However, we believe that this could not have affected the measurement of knowledge. Another limitation was the low percentage of surveyed health professionals working at the hospital and in the Intensive Care Unit; in addition, we could not survey another group of health professionals who were working in more complex health institutions. Therefore, we cannot infer their level of KAP.

In conclusion, being married, having a Master’s degree, and working more than nine hours a day were associated with a low level of knowledge of COVID-19 in health professionals. Being older than 50, and working at the hospital, were associated with preventive practices. Physical contact with patients with COVID-19 was associated with the report of negative attitudes towards COVID-19. We recommend that universities and health institutions incorporate comprehensive training programs that seek to improve knowledge and promote preventive measures against COVID-19.

## Data availability

### Underlying data

Zenodo: Factors associated with knowledge, attitudes and preventive practices towards COVID-19 in health care professionals in Lima, Peru https://doi.org/10.5281/zenodo.4780623. ^46^

Data are available under the terms of the Creative Commons Attribution 4.0 International license (CC-BY 4.0).

## References

[1] Elkholy AA Grant R Assiri AM : MERS-CoV infection among healthcare workers and risk factors for death: Retrospective analysis of all laboratory-confirmed cases reported to WHO from 2012 to 2 June 2018. *J Infect Public Health.* 2020;13:418–422. 10.1016/j.jiph.2019.04.011 31056437 PMC7102841

[2] Bhat M Ghali P Deschenes M : Hepatitis B and the Infected Health Care Worker: Public Safety at What Cost? *Can J Gastroenterol.* 2012;26:257–260. 10.1155/2012/348240 22590698 PMC3352840

[3] Hunter JC Nguyen D Aden B : Transmission of Middle East Respiratory Syndrome Coronavirus Infections in Healthcare Settings, Abu Dhabi. *Emerg Infect Dis.* 2016;22:647–656. 10.3201/eid2204.151615 26981708 PMC4806977

[4] Weinstein RA Bridges CB Kuehnert MJ : Transmission of Influenza: Implications for Control in Health Care Settings. *Clin Infect Dis.* 2003;37:1094–1101. 10.1086/378292 14523774

[5] Wang J Zhou M Liu F : Reasons for healthcare workers becoming infected with novel coronavirus disease 2019 (COVID-19) in China. *J Hosp Infect.* 2020;105:100–101. 10.1016/j.jhin.2020.03.002 32147406 PMC7134479

[6] Schwartz J King C-C Yen M-Y : Protecting Healthcare Workers During the Coronavirus Disease 2019 (COVID-19) Outbreak: Lessons From Taiwan’s Severe Acute Respiratory Syndrome Response. *Clin Infect Dis.* 2020;71:858–860. 10.1093/cid/ciaa255 32166318 PMC7108122

[7] Ajilore K Atakiti I Onyenankey K : College students’ knowledge, attitudes and adherence to public service announcements on Ebola in Nigeria: Suggestions for improving future Ebola prevention education programmes. *Health Education J.* 2017;76:648–660. 10.1177/0017896917710969

[8] Tachfouti N Slama K Berraho M : The impact of knowledge and attitudes on adherence to tuberculosis treatment: a case-control study in a Moroccan region. *Pan Afr Med J.* 2012;12:52. 22937192 PMC3428172

[9] Person B Sy F Holton K : National Center for Inectious Diseases SCOT. Fear and stigma: the epidemic within the SARS outbreak. *Emerg Infect Dis.* 2004;10:358–363. 10.3201/eid1002.030750 15030713 PMC3322940

[10] Tao N : An analysis on reasons of SARS-induced psychological panic among students. *Journal of Anhui Institute of Education.* 2003;21:78–79.

[11] Zhang M Zhou M Tang F : Knowledge, attitude, and practice regarding COVID-19 among healthcare workers in Henan, China. *J Hosp Infect.* 2020;105:183–187. 10.1016/j.jhin.2020.04.012 32278701 PMC7194961

[12] Giao H Han NT Van Khanh T : Knowledge and attitude toward COVID-19 among healthcare workers at District 2 Hospital, Ho Chi Minh City. *Asian Pac J Trop Med.* 2020;13:260–265. 10.4103/1995-7645.280396

[13] Modi PD Nair G Uppe A : COVID-19 Awareness Among Healthcare Students and Professionals in Mumbai Metropolitan Region: A Questionnaire-Based Survey. *Cureus.* 2020;12. 10.7759/cureus.7514 32377462 PMC7198075

[14] Saqlain M Munir MM Rehman SU : Knowledge, attitude, practice and perceived barriers among healthcare workers regarding COVID-19: A cross-sectional survey from Pakistan. *J Hosp Infect.* 2020;105:419–423. 10.1016/j.jhin.2020.05.007 32437822 PMC7211584

[15] Olum R Chekwech G Wekha G : Coronavirus Disease-2019: Knowledge, Attitude, and Practices of Health Care Workers at Makerere University Teaching Hospitals, Uganda. *Front Public Health.* 2020;8:181. 10.3389/fpubh.2020.00181 32426320 PMC7204940

[16] Nepal R Sapkota K Adhikari K : Knowledge, attitude and practice regarding COVID-19 among healthcare workers in Chitwan, Nepal. *J Hosp Infect.* 2020;105:183–187. 10.1016/j.jhin.2020.04.012 32278701 PMC7194961

[17] World Health Organization: Emerging respiratory viruses, including COVID-19: methods for detection, prevention, response, and control. 2020. [2020-02-01]. Reference Source 10.1093/jacamr/dlaa055 PMC7454573 34192251

[18] Bhagavathula AS Aldhaleei WA Rahmani J : Knowledge and Perceptions of COVID-19 Among Health Care Workers: Cross-Sectional Study. *JMIR Public Heal Surveill.* 2020 Apr 30 [cited 2020 Jun 29];6(2):e19160. 10.2196/19160 32320381 PMC7193987

[19] García Cadena CH Landero Hernández R González Ramírez GT : *Estadística con SPSS y metodología de la investigación.* México: Trillas 2006. (pp.139–166).

[20] Kim JS Choi JS : Middle East respiratory syndrome–related knowledge, preventive behaviours and risk perception among nursing students during outbreak. *J Clin Nurs.* 2016;25(17-18):2542–9. 10.1111/jocn.13295 27273475 PMC7166634

[21] Zhang M Zhou M Tang F : Knowledge, attitude, and practice regarding COVID-19 among healthcare workers in Henan, China. *J Hosp Infect.* 2020;105(2):183–187. 10.1016/j.jhin.2020.04.012 32278701 PMC7194961

[22] Tsiga-Ahmed FI Amole TG Musa BM : COVID 19: Evaluating the Knowledge, Attitude and Preventive Practices of Healthcare Workers in Northern Nigeria. *Int J MCH AIDS.* 2021;10(1):88–97. 10.21106/ijma.418 33659097 PMC7905433

[23] Perrotta F Corbi G Mazzeo G : COVID-19 and the elderly: insights into pathogenesis and clinical decision-making. *Aging Clin Exp Res.* 2020;32(8):1599–1608. 10.1007/s40520-020-01631-y 32557332 PMC7298699

[24] Otter JA Donskey C Yezli S : Transmission of SARS and MERS coronaviruses and influenza virus in healthcare settings: the possible role of dry surface contamination. *J Hosp Infect.* 2016;92(3):235–250. 10.1016/j.jhin.2015.08.027 26597631 PMC7114921

[25] Naser AY Dahmash EZ Alsairafi ZK : Knowledge and Practices during the COVID-19 Outbreak in the Middle East: A Cross-Sectional Study. *Int J Environ Res Public Health* .2021;18:4699. 10.3390/ijerph18094699 33925024 PMC8125675

[26] Rani M Sharma I Sharma S : Exploring the knowledge, attitude, and practice of health-care professionals on coronavirus (COVID-19) pandemic infection. *J Educ Health Promot.* 2021;10:115. Published 2021 Mar 31. 10.4103/jehp.jehp_728_20 34084862 PMC8150092

[27] Desalegn Z Deyessa N Teka B : Evaluation of COVID-19 related knowledge and preparedness in health professionals at selected health facilities in a resource-limited setting in Addis Ababa, Ethiopia. *PloS one.* 2021;16(2):e0244050. 10.1371/journal.pone.0244050 33566814 PMC7875347

[28] Addis SG Nega AD Miretu DG : Knowledge, attitude and practice of patients with chronic diseases towards COVID-19 pandemic in Dessie town hospitals, Northeast Ethiopia. *Diabetes Metab Syndr.* 2021;15(3):847–856. 10.1016/j.dsx.2021.03.033 33873054 PMC8028688

[29] Silva JS Batista de Carvalho AR Leite HC : Reflexiones sobre los riesgos ocupacionales en trabajadores de salud en tiempos pandémicos por COVID-19. *Rev Cubana Enferm.* 2020 [citado 22 Jun 2021];36(2):[aprox. 0 p.]. Reference Source

[30] Rizki SA Kurniawan J Budimulia P : Knowledge, Attitude, and Practice in Indonesian Health Care Workers Regarding COVID-19. *Asia Pac J Public Health.* 2021:10105395211011017. 10.1177/10105395211011017 33870724

[31] Ramadan M Hasan Z Saleh T : Beyond Knowledge: Evaluating the Practices and Precautionary Measures towards COVID-19 among Medical Doctors in Jordan. *Int J Clin Pract.* 2021:e14122. 10.1111/ijcp.14122 33650228 PMC7995122

[32] Wang J Zhou M Liu F : Reasons for healthcare workers becoming infected with novel coronavirus disease 2019 (COVID-19) in China. *J Hosp Infect.* 2020;105(1):100–101. 10.1016/j.jhin.2020.03.002 32147406 PMC7134479

[33] Verbeek JH Ijaz S Mischke C : Personal protective equipment for preventing highly infectious diseases due to exposure to contaminated body fluids in healthcare staff. *Cochrane Database Syst Rev.* 2016;4:Cd011621. 10.1002/14651858.CD011621.pub2 27093058 PMC10068873

[34] Williams L Rasmussen S Kleczkowski A : Protection motivation theory and social distancing behaviour in response to a simulated infectious disease epidemic. *Psychol Health Med.* 2015;20(7):832–837. 10.1080/13548506.2015.1028946 25835044

[35] Mortada E Abdel-Azeem A Al Showair A : Preventive Behaviors Towards Covid-19 Pandemic Among Healthcare Providers in Saudi Arabia Using the Protection Motivation Theory. *Risk Manag Healthc Policy.* 2021;14:685–694. 10.2147/RMHP.S289837 33628067 PMC7898786

[36] Dantas F : Resultados terapêuticos da homeopatia em pacientes suspeitos ou confirmados de COVID-19 no Brasil: Protocolo para estudo observacional prospectivo. *Resultados terapêuticos da homeopatia em pacientes suspeitos ou confirmados de COVID-19 no Brasil: Protocolo para estudo observacional prospectivo.* 2020. p.46.

[37] McEachan R Taylor N Harrison R : Meta-Analysis of the Reasoned Action Approach (RAA) to Understanding Health Behaviors. *Ann Behav Med.* 2016;50(4):592–612. 10.1007/s12160-016-9798-4 27169555 PMC4933736

[38] The national institute for occupational safety and health NIOSH: Guidelines for Protecting the Safety and Health of Health Care Workers Revision. 2014. Retrieved June 10, 2020. Reference Source

[39] Yesilgul G Cicek H Avci M : Nurses’ knowledge levels and perceptions regarding occupational risks and hazards. *Int J Caring Sci.* 2018;11(2):1117–1124.

[40] Zhang M Zhou M Tang F : Knowledge, attitude, and practice regarding COVID-19 among healthcare workers in Henan, China. *J Hosp Infect.* 2020;105(2):183–187. 10.1016/j.jhin.2020.04.012 32278701 PMC7194961

[41] Maleki S Najafi F Farhadi K : Knowledge, attitude and behavior of health care workers in the prevention of COVID-19. 2020.

[42] Abdel Wahed WY Hefzy EM Ahmed MI : Assessment of Knowledge, Attitudes, and Perception of Health Care Workers Regarding COVID-19, A Cross-Sectional Study from Egypt. *J Community Health.* 2020;45(6):1242–1251. 10.1007/s10900-020-00882-0 32638199 PMC7340762

[43] Abdelhafiz AS Mohammed Z Ibrahim ME : Knowledge, Perceptions, and Attitude of Egyptians Towards the Novel Coronavirus Disease (COVID-19). *J Community Health.* 2020;45(5):881–890. 10.1007/s10900-020-00827-7 32318986 PMC7173684

[44] Maude RR Jongdeepaisal M Skuntaniyom S : Improving knowledge, attitudes and practice to prevent COVID-19 transmission in healthcare workers and the public in Thailand. *BMC Public Health.* 2021;21(1):749. 10.1186/s12889-021-10768-y 33865342 PMC8053080

[45] Bonilla-Asalde CA Rivera-Lozada IC Bonilla-Pizarro DN : Health sciences students’ competencies in addressing COVID 19: The challenge of returning to clinical practice. *Pakistan J Med Heal Sci.* 2020;14(3):1005–1012.

[46] Lozada OR Bonilla CA : Factors associated with knowledge, attitudes and preventive practices towards COVID-19 in health care professionals in Lima, Peru. 2021. 10.5281/zenodo.4780623 PMC8567687 34804498

