# Supplementary material for: Factors associated with knowledge, attitudes and preventive practices towards COVID-19 in health care professionals in Lima, Peru
Source: F1000Res. 2021 Nov 10;10:582. Originally published 2021 Jul 16. [Version 3] doi: 10.12688/f1000research.53689.3 (PMC8567687; doi:10.12688/f1000research.53689.3)
Supplement: Peer review history

# Peer review: Reviewer response for version 2

**DOI:** 10.5256/f1000research.78038.r97525

**Published:** 2021-11-03

**Authors:** Rubia Consuelo Cobo Rendón

**Affiliations:** 1 Laboratorio de Investigación e Innovación educativa, IDECLAB, Dirección de Docencia, Universidad de Concepción, Concepción, Chile

The authors have made the modifications suggested in the previous version, which has substantially improved the quality of the manuscript. I believe it meets the criteria for approval.

Is the work clearly and accurately presented and does it cite the current literature?

Yes

If applicable, is the statistical analysis and its interpretation appropriate?

I cannot comment. A qualified statistician is required.

Are all the source data underlying the results available to ensure full reproducibility?

Yes

Is the study design appropriate and is the work technically sound?

Yes

Are the conclusions drawn adequately supported by the results?

Yes

Are sufficient details of methods and analysis provided to allow replication by others?

Yes

Reviewer Expertise:

Psychology, well-being and health

I confirm that I have read this submission and believe that I have an appropriate level of expertise to confirm that it is of an acceptable scientific standard.

# Peer review: Reviewer response for version 2

**DOI:** 10.5256/f1000research.78038.r97526

**Published:** 2021-10-25

**Authors:** Jaime A. Yanez

**Affiliations:** 1 Facultad de Educación, Carrera de Educación y Gestión del Aprendizaje, Universidad Peruana de Ciencias Aplicadas, Lima, Peru

**ABSTRACT**:

1. A minor change, please change IC for CI. I understand that IC means intervalo de confianza in Spanish, but CI (confidence interval) should be used.

The manuscript should be approved.

Is the work clearly and accurately presented and does it cite the current literature?

Partly

If applicable, is the statistical analysis and its interpretation appropriate?

Partly

Are all the source data underlying the results available to ensure full reproducibility?

Partly

Is the study design appropriate and is the work technically sound?

Partly

Are the conclusions drawn adequately supported by the results?

Partly

Are sufficient details of methods and analysis provided to allow replication by others?

Partly

Reviewer Expertise:

COVID-19, epidemiology, pharmacology, toxicology, drug development, mental health

I confirm that I have read this submission and believe that I have an appropriate level of expertise to confirm that it is of an acceptable scientific standard.

# Response

**Published:** 2021-10-31

**Authors:** ORIANA RIVERA LOZADA

**Affiliations:** UNIVERSIDAD PRIVADA NORBERT WIENER, Peru

Dear Reviewers,

 The observation survey has already been carried out in the abstract (the IC was changed to the IC) and it was sent to the journal for incorporation.

 Thank you very much for your comments.

**Abstract**

**Background**: Nowadays, we are facing a disease caused by SARS-CoV- 2, known globally as COVID-19, which is considered a threat to global health due to its high contagiousness and rapid spread.

**Methods**: Analytical cross-sectional study in 302 health professionals. An online questionnaire consisting of questions about knowledge, attitudes and practices (KAP) towards COVID-19 was applied. Socio- demographic, occupational and comorbidities factors were explored. Simple and multiple logistic regression models were used to identify factors associated with KAP.

**Results**: Of the total, 25.2%, 31.5% and 37.4% had high levels of knowledge, preventive practices and risk perception attitudes respectively. Being married aOR=6.75 CI(1.46-31.2) p=0.014, having a master's degree aOR=0.4, CI(0.21-0.80) p=0.009, having a working day with less than ten hours ORa=0.49 CI(0.25-0.95) p=0.036 and obesity aOR=0.38 CI (0.15-0.95) p=0.039 were associated with a low level of knowledge of COVID-19. The variables associated with preventive practices were being over the age of 50 aOR=0.52 CI(0.27-0.98) p=0.007, working in the hospitalization area aOR=1.86 CI(1.08-3.18) p=0.018 and having comorbidities such as arterial hypertension aOR=0.28 CI(0.081-0.99) p=0.02 and obesity aOR=0.35 CI(0.14-0.83) p=0.019. In relation to negative attitudes towards COVID-19, it was found that physical contact with patients with a confirmed diagnosis aOR=1.84 CI (1.14-2.97) p=0.006 and having asthma aOR=2.13 CI(1.081-4.22) p=0.029 were associated with these attitudes. **Conclusion**: Our study revealed that health professionals have an insufficient level of knowledge of COVID-19. This is why we recommend implementing strategies such as health literacy programs among health care workers. Thus, they can help develop positive

# Peer review: Reviewer response for version 1

**DOI:** 10.5256/f1000research.57103.r89964

**Published:** 2021-08-11

**Authors:** Jaime A. Yanez

**Affiliations:** 1 Facultad de Educación, Carrera de Educación y Gestión del Aprendizaje, Universidad Peruana de Ciencias Aplicadas, Lima, Peru

**ABSTRACT**

1. It is customary to report not only the aOR but also the CI, as well as the p-value, please include.

**INTRODUCTION**

1. It feels short. There is a need to mention what has been studied and published so far about COVID-19 in Peru and there are plenty of papers related to COVID-19 in Peru. For instance, there is no mention of the effects the lack of KAP has already caused in Peru. Examples of some studies include:
  - Infodemic ^1^^,^^2^^,^^3^
  - Self-medication ^4^^,^^5^
  - Medicinal plants use ^6^^,^^7^^,^^8^^,^^9^
  - Use of unproven treatments such as chlorine dioxide ^6^^,^^10^
  - Issues in children with the development of MIS-C ^11^^,^^12^^,^^13^^,^^14^
  - Impact in mental health ^15^^,^^16^^,^^17^^,^^18^^,^^19^
  - Technostress ^20^
  - Issues in adequate implementation of public health measures ^21^^,^^22^^,^^23^
  - Furthermore, Peru is leading some interesting aspects compared to Latin America in the implementation of telemedicine ^24^^,^^25^^,^^26^^,^^27^

**METHODS**

1. Please indicate in the Study setting and design the exact dates that the survey was available for responses.
2. What exclusion criteria were used?
3. Please deepen the study population and size. It is not clear if 302 respondents were a sufficient sample size for this study. Also, indicate what power were the authors looking for.
4. It is really hard to follow how big was the instrument. Please include the full survey as an Annex to understand the instrument and for other researchers to replicate it.

**RESULTS**

1. Change the word "epidemiological" for "demographic" in the following sentence: Regarding epidemiological variables...
2. Please include the demographic data in a table, which is customary for cross-sectional studies.
3. Clarify the value that represented the 75th percentile in this sentence: In the case of preventive practices, 31.5% (n = 95) obtained scores above the 75th percentile. Please include cut-off values for the surveys used.
4. The same comment as above for the level of risk perception.
5. Please include the p-value for the results of the bivariate analysis.
6. Table 3, the constants are not necessary to be reported, nor the degrees of freedom for a dichotomous variable.

Is the work clearly and accurately presented and does it cite the current literature?

Partly

If applicable, is the statistical analysis and its interpretation appropriate?

Partly

Are all the source data underlying the results available to ensure full reproducibility?

Partly

Is the study design appropriate and is the work technically sound?

Partly

Are the conclusions drawn adequately supported by the results?

Partly

Are sufficient details of methods and analysis provided to allow replication by others?

Partly

Reviewer Expertise:

COVID-19, epidemiology, pharmacology, toxicology, drug development, mental health

I confirm that I have read this submission and believe that I have an appropriate level of expertise to confirm that it is of an acceptable scientific standard, however I have significant reservations, as outlined above.

## References

[1] : The Peru Approach against the COVID-19 Infodemic: Insights and Strategies. *Am J Trop Med Hyg* .103(2) : 10.4269/ajtmh.20-0536 583-586 10.4269/ajtmh.20-0536 32500853PMC7410469

[2] : [Infodemic: fake news and COVID-19 mortality trends in six Latin American countriesInfodemia: notícias falsas e tendências na mortalidade por COVID-19 em seis países da América Latina]. *Rev Panam Salud Publica* .2021;45: 10.26633/RPSP.2021.44 e44 10.26633/RPSP.2021.44 34007261PMC8118087

[3] : [COVID-19 infodemic management strategies in South AmericaEstrategias adoptadas en América del Sur para la gestión de la infodemia relacionada con la COVID-19]. *Rev Panam Salud Publica* .2021;45: 10.26633/RPSP.2021.43 e43 10.26633/RPSP.2021.43 33995518PMC8111039

[4] : Self-medication practices during the COVID-19 pandemic among the adult population in Peru: A cross-sectional survey. *Saudi Pharm J* .2021;29(1) : 10.1016/j.jsps.2020.12.001 1-11 10.1016/j.jsps.2020.12.001 33519270PMC7832015

[5] : Self-medication in times of COVID-19. A perspective from Peru. *Gaceta de M�xico* .2021;157(1) : 10.24875/GMM.M21000526 10.24875/GMM.M21000526 34125806

[6] : Prescription, over-the-counter (OTC), herbal, and other treatments and preventive uses for COVID-19.2021; 10.1016/B978-0-323-85780-2.00001-9 379-416 10.1016/B978-0-323-85780-2.00001-9

[7] : Use of medicinal plants for COVID-19 prevention and respiratory symptom treatment during the pandemic in Cusco, Peru: A cross-sectional survey. *medRxiv* .2021; 10.1101/2021.05.26.21257890 10.1101/2021.05.26.21257890 PMC845747934550994

[8] : Uncaria tomentosa (cat's claw): a promising herbal medicine against SARS-CoV-2/ACE-2 junction and SARS-CoV-2 spike protein based on molecular modeling. *J Biomol Struct Dyn* .2020; 10.1080/07391102.2020.1837676 1-17 10.1080/07391102.2020.1837676 33118480PMC7657399

[9] : Investigating Potential Inhibitory Effect of Uncaria tomentosa (Cat's Claw) against the Main Protease 3CLpro of SARS-CoV-2 by Molecular Modeling. *Evid Based Complement Alternat Med* .2020;2020: 10.1155/2020/4932572 4932572 10.1155/2020/4932572 33029165PMC7532411

[10] : Chlorine dioxide and chlorine derivatives for the prevention or treatment of COVID-19: a systematic review. *Rev Peru Med Exp Salud Publica* .37(4) : 10.17843/rpmesp.2020.374.6330 605-610 10.17843/rpmesp.2020.374.6330 33566898

[11] : Covid-19 in Peru: from supervised walks for children to the first case of Kawasaki-like syndrome. *BMJ* .2020; 10.1136/bmj.m2418 10.1136/bmj.m2418 32571770

[12] : High incidence of stroke and mortality in pediatric critical care patients with COVID-19 in Peru. *Pediatr Res* .2021; 10.1038/s41390-021-01547-x 10.1038/s41390-021-01547-x 33941862PMC8090521

[13] : Acute Abdomen and Appendicitis in 1010 Pediatric Patients With COVID-19 or MIS-C: A Multinational Experience from Latin America. *Pediatr Infect Dis J* .2021; 10.1097/INF.0000000000003240 10.1097/INF.0000000000003240 34260501PMC8443421

[14] : [Cross-sectional study of the clinical characteristics and outcomes of children hospitalized with COVID-19 in Lima, Peru]. *Medwave* .2021;21(1) : 10.5867/medwave.2021.01.8107 e8107 10.5867/medwave.2021.01.8107 33617519

[15] : Anxiety, Distress, and Turnover Intention of Healthcare Workers in Peru by Their Distance to the Epicenter during the COVID-19 Crisis. *Am J Trop Med Hyg* .103(4) : 10.4269/ajtmh.20-0800 1614-1620 10.4269/ajtmh.20-0800 32815512PMC7543861

[16] : Hospitality workers’ COVID-19 risk perception and depression: A contingent model based on transactional theory of stress model. *International Journal of Hospitality Management* .2021;95: 10.1016/j.ijhm.2021.102935 10.1016/j.ijhm.2021.102935 PMC975683236540684

[17] : Factors associated with mental health in Peruvian medical students during the COVID-19 pandemic: a multicentre quantitative study. *Rev Colomb Psiquiatr* .2021; 10.1016/j.rcp.2021.06.002 10.1016/j.rcp.2021.06.002 34275600PMC9186139

[18] : Self-reported psychological problems and coping strategies: a web-based study in Peruvian population during COVID-19 pandemic. *BMC Psychiatry* .2021;21(1) : 10.1186/s12888-021-03326-8 10.1186/s12888-021-03326-8 34256718PMC8276225

[19] : Explanatory Model of Perceived Stress in the General Population: A Cross-Sectional Study in Peru During the COVID-19 Context. *Front Psychol* .2021;12: 10.3389/fpsyg.2021.673945 673945 10.3389/fpsyg.2021.673945 34248770PMC8264254

[20] : Influence of Technostress on Academic Performance of University Medicine Students in Peru during the COVID-19 Pandemic. *Sustainability* .2021;13(16) : 10.3390/su13168949 10.3390/su13168949

[21] : Tratamiento de la COVID-19 en Perú y Bolivia y los riesgos de la automedicación. *Revista Cubana de Farmacia* .2020;53(2) : Reference source

[22] : Peru to punish bending of clinical-trial rules. *Nature* .2021;595(7869) : 10.1038/d41586-021-02056-x 10.1038/d41586-021-02056-x 34316051

[23] : Adverse events related to COVID-19 vaccines: the need to strengthen pharmacovigilance monitoring systems. *Drugs Ther Perspect* .2021; 10.1007/s40267-021-00852-z 1-7 10.1007/s40267-021-00852-z 34366660PMC8327058

[24] : Telemedicine in Peru as a Result of the COVID-19 Pandemic: Perspective from a Country with Limited Internet Access. *Am J Trop Med Hyg* .2021; 10.4269/ajtmh.21-0255 10.4269/ajtmh.21-0255 33999847PMC8274764

[25] : COVID-19 reveals the urgent need to strengthen nursing informatics competencies: a view from Peru. *Inform Health Soc Care* .2021;46(3) : 10.1080/17538157.2021.1941974 229-233 10.1080/17538157.2021.1941974 34292802

[26] : La Telemedicina como herramienta para enfrentar la atención de pacientes durante el contexto de la COVID-19. *Atención Primaria* .2021;53(7) : 10.1016/j.aprim.2021.102061 10.1016/j.aprim.2021.102061 PMC799806033865008

[27] : Telemedicina en el currículo médico para la atención a pacientes geriátricos tras la COVID-19. *Revista Española de Geriatría y Gerontología* .2021;56(2) : 10.1016/j.regg.2021.01.004 122-123 10.1016/j.regg.2021.01.004 PMC786287933608165

# Response

**Published:** 2021-09-28

**Authors:** ORIANA RIVERA LOZADA

**Affiliations:** UNIVERSIDAD PRIVADA NORBERT WIENER, Peru

ABSTRACT

 It is customary to report not only the aOR but also the CI, as well as the p-value, please include.

**Author response: They were added in the abstract.**

 INTRODUCTION

 It feels short. There is a need to mention what has been studied and published so far about COVID-19 in Peru and there are plenty of papers related to COVID-19 in Peru. For instance, there is no mention of the effects the lack of KAP has already caused in Peru. Examples of some studies include:

- Infodemic ^1,2,3^
- Self-medication ^4,5^
- Medicinal plants use ^6,7,8,9^
- Use of unproven treatments such as chlorine dioxide ^6,10^
- Issues in children with the development of MIS-C ^11,12,13,14^
- Impact in mental health ^15,16,17,18,19^
- Technostress ^20^
- Issues in adequate implementation of public health measures ^21,22,23^
- Furthermore, Peru is leading some interesting aspects compared to Latin America in the implementation of telemedicine ^24,25,26,27^

**Author response: The introduction was modified, taking into account the references suggested by the reviewer**.

 METHODS

 Please indicate in the Study setting and design the exact dates that the survey was available for responses.

**Author response: The period was specified in the Study setting and design section (01 August-15 December 2020)**.

 What exclusion criteria were used?

**Author response:  We explained this in the Study population and size section: “To achieve the objectives of our study, we used the following selection criteria: health professionals working at a health center in Lima-Callao who, in addition, were teaching at the Faculty of Health Sciences or at the Graduate School of Norbert Wiener University. The exclusion criteria considered work at the university for less than one year.”**

 Please deepen the study population and size. It is not clear if 302 respondents were a sufficient sample size for this study. Also, indicate what power were the authors looking for.

**Author response: It was specified in the Study population and size section “The sample size was calculated probabilistically in two stages. In the first stage, we determined the sample. For this study, the sample frame was 672 teachers, who were registered in the database of the human resources area of the university. For the calculation of the sample, an expected 50% prevalence was considered, using a confidence level of 97% and an error percentage of 3% and we could obtain an estimated sample of 277 participants. In the second stage, the number of sample elements in each of the strata was calculated through proportional allocation. This was done by dividing the sample size by the population size and then multiplying by the size of each of the strata (APS). Thus, the size of the stratum was directly proportional to the sample size.**

**Sampling was performed through random selection of participants, since the list of health professionals from the academic professional schools (APS) that were part of the study population was available.”**

 It is really hard to follow how big was the instrument. Please include the full survey as an Annex to understand the instrument and for other researchers to replicate it.

**Author response: It was added in annexes.**

 RESULTS

 Change the word "epidemiological" for "demographic" in the following sentence: Regarding epidemiological variables

**Author response: It was corrected:**

**“Information about 302 health professionals who were providing healthcare services during the period August-December** **2020 was obtained. Regarding demographic variables, 64.9% were female and the median age was 46 years old (IQR 42-51)”.**

 Please include the demographic data in a table, which is customary for cross-sectional studies.

**Author response: We added Table 1 with the demographic data of the population.**

 Clarify the value that represented the 75th percentile in this sentence: In the case of preventive practices, 31.5% (n = 95) obtained scores above the 75th percentile. Please include cut-off values for the surveys used.

 The same comment as above for the level of risk perception.

**Author response: The values were included.**

**In the case of level of knowledge, 25.2% showed scores ≥ the 75th percentile, where the cut-off point was in the scores greater than or equal to 34, a parameter that permitted us to establish a high level of knowledge of COVID-19.  The responses with the lowest scores were those related to the severity of the disease according to age groups (42.7%), time of subsistence of the virus (50%), and the need for specialized hospitals to care for suspected or diagnosed infection (55.6%).**

**In the case of preventive practices, 31.5% (n = 95) obtained scores above the 75th percentile ( *the cut-off point was the scores greater than or equal to 11*), which indicated a high level.   A low level of practices was identified, among them, we had the use of disposable gloves in the workplace (45.0%), the use of disposable gowns (42.1%), the use of personal protective equipment (PPE) (25.2%), and the decontamination of surfaces (7.7%).**

**The level of risk perception attitudes towards COVID 19 was analyzed with an inverse scale and we could determine the frequency of low levels of manifestation of negative attitudes (fear of contagion, fear that family members could contract the disease, fear that personal protective equipment could not work, fear of death) such as confidence, fear, concern, and physical and mental fatigue. A total of 37.4% (n = 113) had scores above the 75th percentile (cut-off point greater than or equal to 5), with a predominance of fear of becoming infected (49.7%), returning home, and infecting the family (45%) and fear of dying from COVID 19 (49.7%).**

 Please include the p-value for the results of the bivariate analysis

**Author response: nIt was included.**

 Table 3, the constants are not necessary to be reported, nor the degrees of freedom for a dichotomous variable.

**Author response: We took the constants and the degrees of freedom out of the table.**

# Peer review: Reviewer response for version 1

**DOI:** 10.5256/f1000research.57103.r89965

**Published:** 2021-08-03

**Authors:** Rubia Consuelo Cobo Rendón

**Affiliations:** 1 Laboratorio de Investigación e Innovación educativa, IDECLAB, Dirección de Docencia, Universidad de Concepción, Concepción, Chile

This is a significant study as this manuscript contributes to the identification of factors associated with knowledge, attitudes and preventive practices towards COVID-19 in health care professionals in Lima, Peru. The findings in the study are of value for further research and institutional action associated with the results found in Peru and Latin America. 

 Following the review, I describe the following suggestions:

**Abstract:**

- It is suggested to improve the presentation of the conclusions, they look like a repetition of the results.
- The implications of the findings found could be deepened.

**Introduction:**

- Presentation relevant to the reality of the context studied. It is suggested to deepen the ideas on the justification of the research, with the information presented in the paragraph: "Hence, low levels of knowledge, attitudes and practices (KAP) in regard to the implementation of preventive measures against the disease ^2-6,9^ might cause serious public health problems, since health personnel must assume responsibility for care and control of the pandemic. ^7-10^". 
- Include the objective of the study in the last paragraph. 

**Method: **

- It is important to describe the health context (pandemic) in which the study participants presented themselves at the time of answering the questionnaire. 

**Study procedure and tool:**

- Include the category "Pandemic-associated factors before" the phrase: "relatives with suspected COVID-19 and physical contact with COVID-19 patients)", e.g. "relatives with suspected COVID-19 and physical contact with COVID-19 patients". 

The Results and Discussion are well described. Punctuation and spelling throughout the manuscript should be checked. 

 Reviewer suggestion is appreciated. Best wishes for future work.

Is the work clearly and accurately presented and does it cite the current literature?

Yes

If applicable, is the statistical analysis and its interpretation appropriate?

I cannot comment. A qualified statistician is required.

Are all the source data underlying the results available to ensure full reproducibility?

Yes

Is the study design appropriate and is the work technically sound?

Yes

Are the conclusions drawn adequately supported by the results?

Yes

Are sufficient details of methods and analysis provided to allow replication by others?

Yes

Reviewer Expertise:

Psychology, well-being and health

I confirm that I have read this submission and believe that I have an appropriate level of expertise to confirm that it is of an acceptable scientific standard, however I have significant reservations, as outlined above.

# Response

**Published:** 2021-09-28

**Authors:** ORIANA RIVERA LOZADA

**Affiliations:** UNIVERSIDAD PRIVADA NORBERT WIENER, Peru

This is a significant study as this manuscript contributes to the identification of factors associated with knowledge, attitudes and preventive practices towards COVID-19 in health care professionals in Lima, Peru. The findings in the study are of value for further research and institutional action associated with the results found in Peru and Latin America.

 Following the review, I describe the following suggestions:

 Abstract:

- It is suggested to improve the presentation of the conclusions, they look like a repetition of the results.
- **Author response: The conclusion in the abstract was improved:** **Our study revealed that health professionals have an insufficient level of knowledge of COVID-19. This is why we recommend implementing strategies such as health literacy programs among health care workers. Thus, they can help develop positive attitudes towards the acquisition of self-care habits at work that, in turn, improve their confidence so that health care workers can provide adequate care for their patients and protect themselves.**
- The implications of the findings found could be deepened.
- **Author response: They were deepened in the discussion since we cannot extend much in the abstract.**
- Presentation relevant to the reality of the context studied. It is suggested to deepen the ideas on the justification of the research, with the information presented in the paragraph: "Hence, low levels of knowledge, attitudes and practices (KAP) in regard to the implementation of preventive measures against the disease2-6,9 might cause serious public health problems, since health personnel must assume responsibility for care and control of the pandemic.7-10".
- Include the objective of the study in the last paragraph.
- **Author response: The introduction was enhanced, taking into account the suggestions**.

Method:

- It is important to describe the health context (pandemic) in which the study participants presented themselves at the time of answering the questionnaire.
- **Author response: It was added.**

Study procedure and tool:

- Include the category "Pandemic-associated factors before" the phrase: "relatives with suspected COVID-19 and physical contact with COVID-19 patients)", e.g. "relatives with suspected COVID-19 and physical contact with COVID-19 patients".
- **Author response: It was added.**
